# A pharmacological characterization of *Cannabis sativa* chemovar extracts

**DOI:** 10.1186/s42238-020-00026-0

**Published:** 2020-05-11

**Authors:** Alykhan Devsi, Brett Kiyota, Theophile Ouellette, Andrew P. Hegle, Ricardo E. Rivera-Acevedo, Jasper Wong, Ying Dong, Michael K. Pugsley, Timothy Fung

**Affiliations:** Department of Pharmacology & Toxicology, Cannevert Therapeutics Ltd., 2176 Health Sciences Mall, Vancouver, BC V6T 1Z3 Canada

**Keywords:** Δ^9^-tetrahydrocannabinol, Cannabidiol, analgesia, mouse, cannabis, Cannabichromene, Cannabigerol, Cannabinol, Tail suspension test, Hot plate test

## Abstract

**Background:**

Cannabis contains Δ^9^-tetrahydrocannabinol (Δ^9^-THC) and cannabidiol (CBD) as the primary constituents responsible for pharmacological activity. However, there are numerous additional chemically-related structures to Δ^9^–THC and CBD that are pharmacologically active and may influence the pharmacological properties of Δ^9^-THC and CBD. This study chemically characterized the cannabinoid constituents in a series of cannabis chemovar extracts and investigated the potential cannabinoid entourage effect in two behavioral assays.

**Methods:**

Six chemovar extracts were compared to pure Δ^9^-THC, CBD and morphine for effects on the following behavioral assays in mice: hot plate and tail suspension. The battery of behavioral tests was conducted post intravenous administration of cannabis chemovar extract. Cannabinoid profiles of extracts were analyzed using high performance liquid chromatography. Cannabis extracts were administered at equal doses of Δ^9^-THC to investigate the role of their cannabinoid profiles in modulating the effects of Δ^9^-THC. Dose response curves were fit using a log[inhibitor] vs response three parameter model and differences between group means were determined using a one-way ANOVA followed by a post hoc test.

**Results:**

Cannabis chemovars tested in this study exhibited substantially different cannabinoid profiles. All chemovars produced dose-dependent immobility in the tail suspension assay and dose-dependent antinociception in the hot plate assay. The maximum antinociceptive effect and ED50 was comparable between cannabis chemovars and Δ^9^-THC. Two cannabis chemovars produced significantly greater immobility in the tail suspension test, with no significant differences in ED50.

**Conclusions:**

Commercially available cannabis chemovars vary widely in cannabinoid content, but when equalized for Δ^9^-THC content, they produce similar behavioral effects with two exceptions. These findings provide only limited support for the entourage hypothesis. Further studies are necessary to characterize the nature of these pharmacological differences between cannabis chemovars and pure Δ^9^-THC.

## Introduction

The pharmacological activity of Δ^9^-tetrahydrocannabinol (Δ^9^-THC), the primary active ingredient of the *Cannabis sativa* L. (*Cannabis spp.* or *cannabis*) plant, mediates its psychoactive and allied physiological effects primarily through activation of the G-protein coupled cannabinoid type 1 (CB_1_). However, Δ^9^-THC also interacts with the type 2 cannabinoid (CB_2_) receptor as well as non-CB receptors (Pertwee 2006; Borgelt et al. 2013; Morales et al. 2017). CB_1_ receptors reside primarily in neuronal tissues and are responsible for the psychotropic actions associated with cannabinoids in cannabis. Conversely, cannabidiol (CBD), the other major component of cannabis, is non-psychoactive, exhibits negative allosteric modulation of the CB_1_ receptor in vitro and displays inverse agonism at the CB_2_ receptor (Pertwee 2006; Borgelt et al. 2013; Morales et al. 2017). In addition to Δ^9^-THC and CBD, the *Cannabis sativa* plant contains over 120 unique cannabinoids, several of which have been demonstrated to possess pharmacological activity (Morales et al. 2017). The antinociceptive effect of Δ^9^-THC has been demonstrated in a variety of non-clinical pharmacological models that includes acute, inflammatory and chronic pain (Robson 2014). With that said, the leading limitation to the use of cannabinoids as potential therapeutic agents are the psychoactive or altered sensorimotor and cognitive properties associated with cannabinoid receptor modulation (Robson 2014; Nissen et al. 2008). However, recently, a liquid formulation of a highly purified plant-derived CBD (Epidiolex®) was approved for use in the US by the FDA for use in two rare genetic forms of childhood epilepsy (Lennox-Gastaut and Dravet’s syndrome) where the drug has shown to produce significantly greater reductions in seizure types and frequency in these patients supporting the therapeutic viability of cannabinoids (O'Connell et al. 2017; Lattanzi et al. 2018).

Analgesia resulting from the use of cannabis or cannabis extracts has, until recently, been primarily mediated by non-clinical drug development (Roques et al. 2012). However, marked safety issues have halted further clinical assessment (Eddleston et al. 2016). Thus, a re-evaluation of cannabis is warranted because of the limited adverse event profile associated with its medical use. It is the pleiotropic efficacy associated with cannabis plant extracts that has conveyed benefits to many diseases including pain, multiple sclerosis, inflammation, epilepsy, anorexia, glaucoma, emesis, cardiovascular disease, cancer, obesity as well as Parkinson’s and Alzheimer’s disease (Kaur et al. 2016). Although these possible therapeutic benefits associated with the use of cannabis are claimed, they are often poorly substantiated and frequently contradictory (Belendiuk et al. 2015). The pleiotropic effects of cannabis may arise from the synergy between different cannabinoids. In addition to Δ^9^-THC, other cannabinoids also exhibit pharmacological effects and can modulate the effects of Δ^9^-THC (Russo and McPartland 2003; Russo 2011). However, it is unclear which compounds are responsible are for the entourage effect and the evidence for the entourage effect is mixed (Bonn-Miller et al. 2018).

With the legalization of medical marijuana for use in Canada (Anon 2018) it is clear that the application of rigorous scientific research and pharmacological evaluation is required to provide a definitive scientific basis for use as a therapeutic drug in these conditions. In these studies, a series of cannabis chemovar extracts were initially chemically characterized for levels of Δ^9^-THC, CBD and related cannabinoids. Each extract was subjected to pharmacological evaluation using a series of validated, non-clinical murine animal models to determine the therapeutic potential of the individual chemovar extracts and whether there is a difference from anticipated effects mediated by Δ^9^-THC on the basis of cannabinoid content. The purpose of this study is to evaluate the extent to which non- Δ^9^-THC cannabinoids might modify the pharmacological effects of Δ^9^-THC.

## Material and methods

### Animals, handling and dosing

Female CD-1 mice 12 weeks of age and weighing 25–30 g were obtained from Charles River Laboratories (Montreal, QC, Canada) and used in all studies. Animals were group housed and acclimatized for at least one week in a controlled environment at 23–27 °C, 50 ± 20% relative humidity, with a 12 h light/dark cycle. Heat-treated hardwood shavings were used for bedding. All animals were fed a certified laboratory rodent chow (Harlan 2018C Certified Global Rodent Diet, Indianapolis, IN) ad libitum and were permitted free access to tap water. Animals were individually marked on the tail with an indelible marker and allowed to adjust to the change in environment for a minimum of one hour before being randomly assigned to the study.

Cannabis chemovar extracts were administered intravenously (i.v.) and were solubilized in an optimized vehicle solution (1,1,18 ethanol, polyoxyl 35 castor oil, 0.9% saline). Using the concentrations obtained from HPLC analysis, extract concentrations were determined and solutions were prepared by diluting the extracts immediately prior to injection at a dose volume of 1.0 ml/kg body weight (Diehl et al. 2001). Either control vehicle solution, cannabis chemovar extract solution or the positive control drugs morphine (PubChem CID: 5288826), ≥98% Δ^9^-THC (PubChem CID: 2978) or ≥ 98% CBD (PubChem CID: 644019) were administered using U100 BD Safety Guide Insulin syringes (BD - Canada, Mississauga, ON, Canada). All injections sites were inspected for dosing integrity. If no bleeding upon needle withdrawal was observed, the animal was subjected to the testing assays.

All studies were performed with female mice using study protocols approved by the University of British Columbia Animal Care Committee. The use of non-clinical models for such purposes have been extensively addressed and justified in vivo (Curtis et al. 1987). The study design and animal ethics conform with ARRIVE (Kilkenny et al. 2010) and guidance on experimental design and analysis (Curtis et al. 2018).

### Chemovar and dosing solution preparation

The *Cannabis sativa* plant generally contains over 120 unique cannabinoids, several of which have been demonstrated to possess pharmacological activity (Morales et al. 2017). However, a vast majority of cannabinoids have not been investigated and it is reasonable to assume that they are also pharmacologically active. Due to shortcomings in knowledge of the activity of minor cannabinoids, the degree to which they interact with different receptor systems and modulate the activity of each other is currently unknown. In light of this, chemovars were selected in an attempt to represent a variety of cannabinoid profiles. Chemovars contained Δ^9^-THC concentrations ranging from 0.45–7.54 mg/ml and varying concentrations of CBD, CBG, CBDV, CBDA, CBN, THCV, and THCA.

The chemical characterization of the chemovar extract constituents showed a consistent profile of identifiable and quantifiable non-Δ^9^-THC cannabinoids in the extracts. For most cannabis chemovar extracts there is limited pharmacological data available regarding efficacy and safety of the non-Δ^9^-THC cannabinoids present in the extracts (Turner et al. 2017). However, these primarily non-psychotropic phytocannabinoids are emerging as possible key constituents that, theoretically, could modulate the pharmacological properties of the cannabis chemovar extract. In the chemovar extracts characterized, cannabichromene (CBC) was the third most abundant cannabinoid. CBC has been shown to have no affinity for either CB_1_ or CB_2_ receptors but rather affects transient receptor potential (TRP) channels (Morales et al. 2017; De Petrocellis et al. 2008) and inhibits the endogenous cannabinoid, anandamide (De Petrocellis et al. 2011). Cannabigerol (CBG) and cannabidivarin (CBDV) were also found in the chemovars at low but detectable levels except in chemovar CTL-X02.H1. Both CBG and CBDV have limited pharmacological profiles, but in the studies that have been conducted, both have some effects mediated via cannabinoid receptors (Morales et al. 2017). Despite these profiles, the levels of these cannabinoids present within the culture extracts do not appear, in totality, to significantly reduce or augment antinociception or effects on waiting behavior associated with the Δ^9^-THC present in the chemovar extracts. This is likely a consequence of the chemovars containing comparatively high concentrations of Δ^9^-THC which is the main cannabinoid focused on by producers and consumers.

All cannabis chemovar extracts and pure ∆^9^-THC tested in these studies were purchased from CanniMed® Ltd. (Saskatoon, SK, Canada). Cannabis chemovars were chosen to represent a range ∆^9^-THC (e.g. ∆^9^-THC [0.45–7.54 mg/ml]) content and other cannabinoid concentrations. Table 2 shows the cannabinoid profile for tested chemovars. CBD was purchased from Echo Pharmaceuticals (Leiden, The Netherlands). Upon receipt, dried, milled plant material with 10 mm grind size and 15% humidity was stored at room temperature (23–27 °C) in light-protected, air tight foil containers (Ware et al. 2015). The liquid cannabis chemovar extract preparations were stored in a freezer at -20 °C and protected from light. All chemovars were freshly prepared by dissolving in the vehicle solution using a serial dilution method for immediate use in all studies. All dosing of cannabis chemovar extracts was based on the quantity of Δ^9^-THC contained within the chemovar preparation to allow for comparisons of activity. Δ^9^-THC levels were measured prior to use in pharmacology studies using high pressure liquid chromatography (HPLC) methods.

### Determination of Δ^9^-THC, CBD and related cannabinoids in chemovars

*Cannabis sativa* extracts were prepared by hexane liquid-liquid extraction of 5 g dry milled cannabis flowering heads. After concentration in a rotary evaporator, the extracted resin was re-suspended in a vehicle solution containing absolute ethanol, 35 castor oil, and 0.9% NaCl in a ratio of 1:1:18, respectively. Cannabinoid concentrations were quantified on a Shimadzu Prominence high-performance liquid chromatography (HPLC) system (Shimadzu Scientific Instruments (SSI), Columbia, MD, USA), using a mobile phase of 3:1 acetonitrile:water + 0.1% formic acid and detected at 220 nm. The method employed is well validated and is a robust and reliable technique for detecting the neutral form of cannabinoids (Mudge et al. 2017).

A calibration curve was constructed for Δ^9^-THC. No peaks were detected at levels above background in the blank control samples. The extracted standard curves ranged from 0.977–500 ng Δ^9^-THC/mL and were included in each sample analyzed for determination of the Δ^9^-THC concentration in cannabis chemovar extract. Those samples containing concentrations that were outside this range were excluded from analysis. Standard curves were linear (r ≥ 0.99), but when not linear, the study samples were excluded from analysis. Similarly, specificity data were generated for each cannabinoid quantified in this study (Table 1). Cross-reactivity is not believed to be an issue as no difference was observed between the sole compound calibration runs performed in our HPLC and those containing purified mixed cannabinoid samples used in this study. As such, cross reactivity was not specifically addressed. Chemovar doses were based on their Δ^9^-THC content to allow for direct comparison with Δ^9^-THC. The cannabis chemovar cannabinoids that were quantified using HPLC included: cannabidivarin acid (CBDVA), cannabidivarin (CBDV, a cannabidiol homolog), cannabidiolic acid (CBDA), cannabigerol, (CBG), cannabidiol (CBD), Δ^9^-tetrahydrocannabivarin (THCV, a homolog of Δ^9^-THC), cannabinol (CBN), (−)-trans-Δ^9^-tetrahydrocannabinol (Δ^9^-THC), cannabichomene (CBC, occurs primarily as cannabichromenic acid (CBCA)) and Δ^9^-tetrahydrocannabinolic acid (THCA, the conjugate base tetrahydrocannabinolate and precursor of Δ^9^-THC). No other cannabinoids were detected using this methodology (Table 2).

**Table 1:** Specificity Data

Analyte	Retention Time	R^2	LOD (mg/mL)	LOQ (mg/mL)
CBDV	2.857	0.999	0.0001	0.001
CBDA	3.483	0.994	0.0005	0.01
CBG	3.748	0.999	0.0001	0.001
CBD	3.95	0.999	0.0001	0.005
THCV	4.126	0.999	0.0001	0.001
CBN	5.289	0.999	0.0001	0.001
Δ^9^-THC	6.407	0.999	0.0001	0.005
CBC	7.718	0.999	0.0001	0.001
THCA	8.189	0.995	0.0005	0.01

*LOD* Limit of detection

*LOQ* Limit of quantification

*CBDVA* Cannabidivarin acid, *CBDV* Cannabidivarin, *CBDA* Cannabidiolic Acid, *CBG* Cannabigerol, *CBD* Cannabidiol, *THCV* Δ^9^-Tetrahydrocannabivarin, *CBN* Cannabinol, *Δ*^*9*^*-THC* Δ^9^-tetrahydrocannabinol (Δ^9^-THC), *CBC* Cannabichomene, *THCA* Δ^9^-Tetrahydrocannabinolic acid

**Table 2:** The concentrations of Δ^9^-THC and CBD derived from each cannabis cultivar extract

Cultivar Sample	Cannabinoid Concentration (mg/ml)												
	Δ^9^-THC	CBD	CBC	CBG	CBDV	CBDA	CBN	THCV	THCA	CBDVA	CBGA	Max dose Δ^9^-THC administered (mg/kg)	Max dose CBD administered (mg/kg)
**CTL-H01.H3**^**c**^	7.54	0.02	0.17	0.20	–	a	0.07	0.06	0.69	–	–	3	0.01
**CTL-H01.H2**	3.23	a	0.06	0.10	a	a	0.06	0.13	a	–	a	6	a
**CTL-P01.H1**	1.98	4.52	0.31	0.02	0.03	0.09	a	a	a	a	b	6	13.7
**CTL-G01.H8**	0.50	6.52	1.39	0.04	0.07	0.93	0.06	a	a	a	b	3	39.1
**CTL-G03.H2**	0.72	5.49	0.72	a	0.01	0.77	a	b	a	a	b	3	22.9
**CTL-X02.H1**	0.45	2.91	0.37	0.107	0.09	1.31	0.041	0.05	0.77	–	–	2	12.9

(−) indicates cannabinoid was not determined in the preparation

^a^*bLOQ* Below level of quantification for the cultivar assay range (0.977–500 ng Δ^9^-THC/mL). Refers to the limit at which the difference between two distinct values can be distinguished using the assay

^b^*bLOD* Below level of detection for cultivar assay range for all cultivars. The lowest quantity of the extract component distinguished from the absence of that substance (i.e., blank value) with a confidence level of 99%

^c^Indicates sample was re-tested

*CBDVA* Cannabidivarin acid, *CBDV* Cannabidivarin, *CBDA* Cannabidiolic Acid, *CBG* Cannabigerol, *CBD* Cannabidiol, *THCV* Δ^9^-Tetrahydrocannabivarin, *CBN* Cannabinol, *Δ*^*9*^*-THC* Δ^9^-tetrahydrocannabinol (Δ^9^-THC), *CBC* Cannabichomene, *THCA* Δ^9^-Tetrahydrocannabinolic acid

### In vivo behavioral assays

The in vivo studies conducted for the cannabis chemovar extracts include the tail suspension and standard hot plate assays. We chose the hot plate assay to study the antinociceptive effects of chemovars in mice and the tail suspension assay to study additional central nervous system effects that may occur at therapeutic doses. Each test was performed on at least 5 mice of each control, positive control drug or cannabis extract dose group.

#### The time course of effect for responses in various assays

The time course profile of pure Δ^9^-THC given intravenously was used as the reference compound and was characterized in all behavioral assays used to evaluate the chemovars. Δ^9^-THC doses were selected based on pilot studies that demonstrated a maximal analgesic effect in the hot plate assay. The effects of pure Δ^9^-THC dosed at 0.3, 1, 3, and 10 mg/kg were examined between 1 and 120 min in both the tail suspension assay. Δ^9^-THC was given at doses of 0.1, 0.3, 1 and 3 mg/kg in the hot plate assay and examined between 1 and 120 mins. Morphine was also evaluated in these same assays at doses of 0.3, 1, 3, and 10 mg/kg over the same time intervals. CBD was additionally evaluated in these same assays at doses of 4, 8 and 16 mg/kg over comparable time intervals. Cannabis chemovar extracts were dosed in terms of Δ^9^-THC to animals in each assay as follows: CTL-H01-H3 (0.1, 0.3, 1 and 3 mg/kg), CTL-H01-H2 (0.1, 0.3, 1, 3, and 6 mg/kg), CTL-P01-H1 (0.1, 0.3, 1, 3 and 6 mg/kg), CTL-G01-H8 (0.03, 0.1, 0.3, 1 and 3 mg/kg), CTL-G03-H2 (0.03, 0.1, 0.3, 1 and 3 mg/kg) or CTL-X02-H1 (0.1, 0.3, 1, and 2 mg/kg).

#### Tail suspension assay

The mouse tail suspension assay is a well characterized acute behavioral testing method. The assay evaluates the effects of drugs on animal immobility and escape-associated behavior to inescapable stress resulting from tail suspension (Cryan et al. 2005). This assay was selected to evaluate the chemovar extracts since it is a sensitive test to low dose pharmacological effects after only administration of a single dose of extract (Steru et al. 1985). Both Δ^9^-THC and the chemovar extracts increased the duration of immobility, or reluctance of animals to maintain escapist behavioral traits, an effect consistent with activation of CB_1_ receptors in the CNS (Cryan et al. 2005). These effects have been associated with antidepressant activity for which the model is used (El-Alfy et al. 2010). The testing period (120 min in duration) allows for characterization of chemovar effects on this variable (Steru et al. 1985). In this assay each mouse (*n* = 5–10) was randomly administered either the cannabis chemovar extract, positive control drug (morphine, CBD and Δ^9^-THC) or vehicle control and immediately suspended by the tail using adhesive tape (applied 2 cm from the tip of the tail) to a darkened wooden box (30 cm × 30 cm × 30 cm). The mice were suspended 35 cm directly above the base of the apparatus. Animal immobility behavior was defined as the lack of movement (i.e., no active behavior) over a 6 min observation period. Each mouse subjected to the test was scored by a trained experimental observer blinded to the administered treatment.

#### Standard hot plate assay

A modification to the original hot plate assay (Eddy and Leimbach 1953) was used in these studies. The hot plate temperature was held at 48 ± 0.5 °C rather than ~ 55 °C since studies have shown that assessing analgesic activity at a reduced temperature provides greater reproducible, quantifiable and dose-related responses (O’Callaghan and Holtzman 1975). For evaluation, each mouse was placed on a custom-built hot plate consisting of an anodized, aluminum plate (30 × 30 cm) which was uniformly heated and surrounded by a 15 cm high Plexiglas enclosure. Mice (*n* = 5–10) were administered doses of either cannabis chemovar extract, the positive control drugs or vehicle. The time taken for the mouse to react was measured from the initial contact with the plate to endpoint defined by either licking the hind paw or jumping in an attempt to escape. Pre-drug effects were recorded at 180, 120 and 60 min before dosing and at a single time point 5–10 min after drug administration. The maximal duration permitted to observe for a reaction was 120 s before removing the animal from the plate. This duration avoided any possible tissue damage to the mouse not responding (Steru et al. 1985). In this assay, a normal reaction time was defined as the average of the individual pre-drug values. Analgesia was evaluated as the mean maximum actual reaction time.

### Statistical analysis

**Fig. 1:**
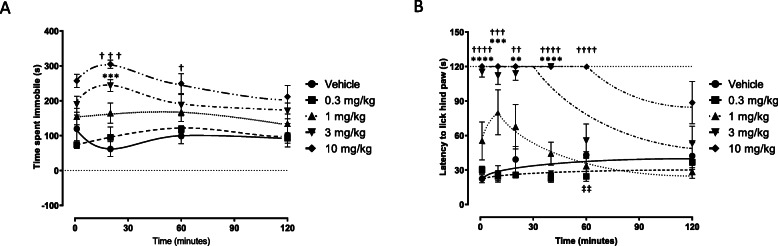
A time-effect profile of pure (≥98%) Δ^9^-THC on immobility time in the tail suspension test (panel **a**). Mice were intravenously dosed with control (vehicle) or 0.3, 1, 3, and 10 mg/kg pure Δ^9^-THC (*n* = 5 per group, 1 animal excluded from 10 mg/kg) and immobility time evaluated for 6 min at various time points at 1, 20, 60, and 120 min. Panel **b** shows the time-effect curve for Δ^9^-THC in the standard hot plate assay. Mice were administered i.v. with vehicle, 0.3, 1, 3, and 10 mg/kg (n = 5 per group, 1 animal excluded from 10 mg/kg). The minimal time spent immobile at time 0 is denoted by the dotted line in panel **a** while the maximal latency to licking of the hind paw is 120 s and denoted by the dotted line in panel **b**. Data is fit using an “eye line of best fit” due to the lack of knowledge regarding the underlying mathematical models. Data are presented as the mean ± SEM for each treatment group. **/††/‡‡ indicates *P* < 0.01, ***/††† indicates *P* < 0.001, ****/†††† indicates *P* < 0.0001. Δ^9^-THC doses were compared to vehicle at the given time point

Analyses of the acquired data proceeded with conduct of systematic analyses for sources of variance and, where necessary, correction for such variance. An ANOVA was performed to identify sources of variance and the statistical significance of group means. Statistical analysis of the dose-response studies used a standard statistical package (GraphPad Prism v.7.0, GraphPad Software, La Jolla, CA). All values are shown as mean ± SEM. Outliers were identified and removed using Grubb’s outlier test. Curve fits for the dose-response curves used the simplest model for reversible drug binding to a receptor where A + R = AR. Using this model, the response is proportional to AR in a non-linear regression model. This equation utilizes a standard slope with a 3parameter configuration based upon the minimal and maximal response profile established for the individual assay. It is recognized that with the imposed cutoff threshold in the hot plate assay, the calculated ED_50_ may not be accurate as the 100% effect in the assay cannot be confirmed to be equal to 100% AR. ED_50_ values reported in this study are defined as an eED_50_, the ethical estimated dose producing a half maximal response. One-way ANOVA followed by a post hoc Tukey’s multiple comparisons test was used to compare baseline data for all extracts. ED50, eED50, and maximum values of chemovars were compared using a one-way ANOVA followed by a post hoc Dunnett’s multiple comparisons test. Tukey’s post hoc multiple comparisons test was used to allow for comparisons between all baseline groups while Dunnett’s multiple comparisons test allowed for comparison of treatment groups to a control group. For all time course figures (Fig. 1), data were fit using an “eye line of best fit” due to inadequate knowledge of the underlying mathematical model. A mixed-effects model followed by a post hoc Dunnett’s multiple comparisons test was used to compare responses between doses at each time point. Statistical significance was defined as *P* < 0.05.

## Results

### Determination of Δ^9^-THC, CBD and related cannabinoids in chemovars

A quantitative evaluation of the principal cannabinoid constituents was conducted for all cannabis chemovars (Table 2). Of the samples tested, chemovar CTL-H01.H3 contained the highest level of Δ^9^-THC (7.54 mg/ml) but the lowest level of CBD (0.02 mg/ml). On the other hand, CTL-X02.H1 had the lowest level of Δ^9^-THC (0.45 mg/ml) but a moderate level of CBD (2.91 mg/ml) compared to other chemovars. Chemovars CTL-P01.H1, CTL-G01.H8, and CTL-G03.H2 had comparable high levels of CBD (4.52–6.52 mg/ml) but low levels of Δ^9^-THC. The other cannabinoids (CBC, CBG, CBDV, CBDA, CBN, THCV and THCA) were determined at variable levels in all chemovars and are shown in Table 2.

### In vivo behavioral assays

#### The time course of effect of pure Δ^9^-THC and morphine in each assay

Determining the time to pharmacological effect is important to ensure that the response is evaluated at an approximate steady-state. When given i.v., the time course profile of pure Δ^9^-THC was characterized in all assays. Figure 1 shows the time-effect profiles of pure Δ^9^-THC given in the tail suspension (A)and hot plate assays (B) for up to 120 mins after dosing. Mixed effects analysis followed by a post hoc Dunnett’s test found significant differences between vehicle (*n* = 5) and 3 mg/kg Δ^9^-THC (n = 5) at 20 min (*P* = 0.0007), vehicle and 10 mg/kg Δ^9^-THC (n = 5, 1 animal excluded) at 20 min (*P* = 0.0002) and 60 min (*P* = 0.0120). In the hot plate assay, the same analysis found significant differences between vehicle (*n* = 5) and 3 mg/kg Δ^9^-THC (n = 5) at 1 min (*P* < 0.0001), 10 min (P = 0.0002), 20 min (*P* = 0.0031), and 40 min (P < 0.0001). Significant differences were also detected between vehicle and 10 mg/kg (n = 5, 1 animal excluded) at 1 min (P < 0.0001), 10 min (*P* < 0.0004), 20 min (*P* = 0.0055), 40 min (P < 0.0001), and 60 min (P < 0.0001). Interestingly, a significant difference was detected between vehicle and 0.3 mg/kg (n = 5) at 60 min (*P* = 0.0274), but this may have an artefact.

Thus, the time course experiments supported the peak-effect being reached at 20 min for the tail suspension assay and 5–10 min for the standard hot plate assay. The effects dissipated to control levels within the 120 min testing period. From these curves, the evaluation of chemovar effects for the tail suspension assay occurred starting 1 min after injection to ensure the peak effect was not missed. In the hot plate assay, effects were evaluated starting 10 min after injection since latency was increased for at least 20 mins.

Since morphine is an analgesic that has been known and used for many decades as a reference compound in non-clinical studies (Kaneto and Nakanishi 1971; Lutfy et al. 1991), the i.v. doses administered were generally of a similar magnitude to a therapeutic dose (Kaneto and Nakanishi 1971). Morphine responses were evaluated over the same time periods as pure Δ^9^-THC. CBD (up to 16 mg/kg, i.v.) was devoid of any time course of effect in the assays evaluated.

#### The dose-response effects of reference compounds (Δ^9^-THC and morphine)

**Fig. 2:**
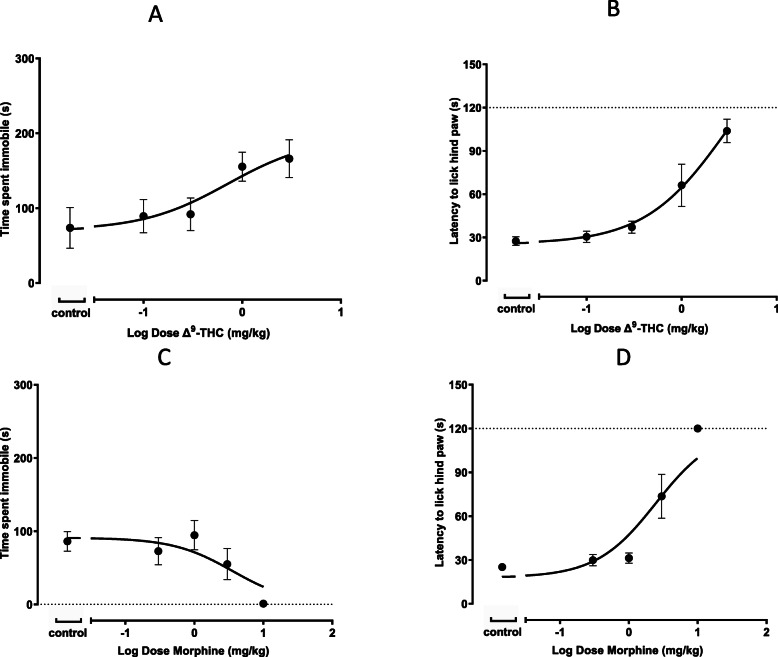
The effects of Δ^9^-THC and morphine in each behavioral assay. Mice were intravenously dosed with control (vehicle) (*n* = 7), 0.3 (n = 7, 1 animal excluded), 1 (n = 7), 3 mg/kg (n = 7) Δ^9^-THC (panel **a**) or control (vehicle) (n = 7), 0.3 (n = 7), 1 (n = 7), 3 (n = 7), or 10 mg/kg (n = 7) morphine (panel **c**) and immobility time in the tail suspension assay was evaluated 20 min post dose. The effects of Δ^9^-THC (n = 7 per group) (panel **b**) and morphine (n = 7 per group) (panel **d**) were evaluated 5–10 min post-dose in the standard hot plate assay. Note that the maximal duration is denoted as 120 s in this assay by the dotted lines (panels **b** and **d**). Data is presented as the mean ± SEM. Data were fit using a 3 parameter log (agonist) vs. response non-linear regression model

The i.v. administration of pure Δ^9^-THC produced the anticipated non-clinical behavioral responses in mice as has been characterized in these assays previously (Huestis 2005; El-Alfy et al. 2010). Administration of pure Δ^9^-THC (*n* = 7 per group) produced analgesia (Fig. 2b) but also reduced activity, as shown in the tail suspension assay, since there was a dose-dependent increase in the time animals spent immobile (*n* = 7 per group, 1 animal excluded from 0.3 mg/kg) (Fig. 2a). The analgesic logED_50_ values are shown in Table 3. Conversely, CBD (up to 16 mg/kg, i.v.) was devoid of any dose-dependent effect in the assays evaluated.

**Table 3:** The EC_50_ values for each cultivar in each behavioral assay

Positive Control	Tail Suspension (s)		Hot Plate Latency (s)	
	^**a**^logED_**50**_ ± SEM (mg/kg)	^**b**^Maximum Effect	^**c**^elogED50 ± SEM (mg/kg)	Maximum Effect
**Morphine**	0.56 ± 0.29		0.39 ± 0.12	
Δ^9^-**THC**	−0.14 ± 0.59	100%	0.03 ± 0.13	100%
**Cultivar Sample**				
**CTL-H01.H3**	−0.37 ± 0.23	151 ± 7%**	−0.31 ± 0.09	69 ± 17%
**CTL-H01.H2**	− 0.13 ± 0.29	118 ± 12%	0.08 ± 0.16	104 ± 12%
**CTL-P01.H1**	–	–	0.03 ± 0.13	106 ± 9%
**CTL-G01.H8**	0.35 ± 1.13	101 ± 17%	0.12 ± 0.10	116 ± 0%^4^
**CTL-G03.H2**	0.14 ± 0.67	112 ± 3%	–	–
**CTL-X02.H1**	−0.48 ± 0.25	146 ± 8%*	−0.37 ± 0.10	111 ± 4%

^a^logED50 values are presented for i.v. dose administration of positive controls or cultivar samples

^b^Maximum effect normalized to the maximum effect of Δ^9^-THC at a dose of 3 mg/kg (with the exception of CTL-X02.H1 compared at 2 mg/kg)

^c^The elogED_50_ is the ethical estimated dose producing a 50% response (see methods for detailed explanation)

^d^All animals reached the ethical cutoff threshold

- Indicates that no ED_50_ value could be determined (see text for details)

* indicates a statistically significant difference (*P* < 0.05) compared to Δ^9^-THC, ** indicates a statistically significant difference (*P* < 0.01) compared to Δ^9^-THC

In contrast, morphine administration to mice produced a dose-dependent, excitatory behavior that included hyperactivity, Straub tail and the stereotypical ‘running fit’ response (Brase et al. 1991; Pacifici et al. 1984). Morphine dose-dependently reduced the time animals spent immobile (n = 7 per group) (Fig. 2c) and prolonged the latency for the time to lick the hind paw in the hot plate assay (n = 7 per group) (Fig. 2d).

#### Effects of cannabis chemovar extracts in the tail suspension assay

**Fig. 3:**
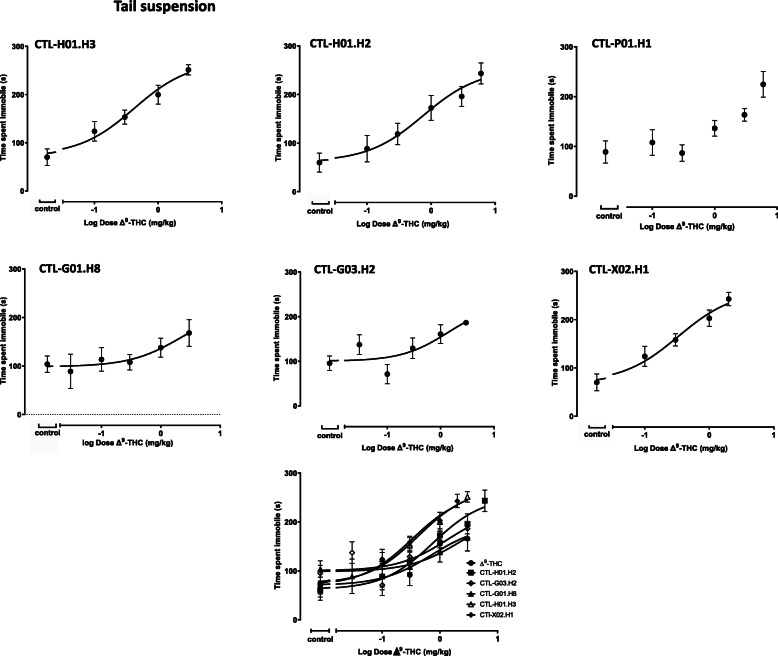
The effects of cannabis cultivar extracts were examined in the tail suspension assay. Mice were intravenously dosed with control (vehicle) or CTL-H01-H3 (0.1, 0.3, 1 and 3 mg/kg) (*n* = 10 per group, 1 animal excluded from 0.01, 0.3, and 1 mg/kg), CTL-H01-H2 (0.1, 0.3, 1, 3 and 6 mg/kg) (*n* = 6 per dose with 1 animal excluded from the 0.1 mg/kg group), CTL-P01-H1 (0.1, 0.3, 1, 3 and 6 mg/kg) (n = 6 per group, 2 animals and 1 animal excluded from the 1 mg/kg and 6 mg/kg groups, respectively), CTL-G01-H8 (0.03, 0.1, 0.3, 1 and 3 mg/kg) (n = 6 per group with 1 animal excluded from 0.03, 0.1, 0.3, and 1 mg/kg), CTL-G03-H2 (0.03, 0.1, 0.3, 1 and 3 mg/kg) (n = 6 per group with 1 animal excluded from 1 mg/kg) or CTL-X02-H1 (0.1, 0.3, 1, and 2 mg/kg) (n = 10 per group, 1 animal excluded from 0.01 and 2 mg/kg) cannabis cultivar extract. The curves have been summarized on the same set of axes to facilitate visual comparisons. Immobility time was evaluated 20 min post dose. Data are presented as the mean ± SEM. Data were fit using a 3 parameter log (agonist) vs. response non-linear regression model. Dosing was based on the Δ^9^-THC content of the cannabis cultivar extract, as analyzed by HPLC

Two cannabis chemovar extracts with different cannabinoid profiles administered i.v. produced a dose-dependent increase in the time animals spent in an immobile posture (Fig. 3). Dose-response curves for chemovars may only be an approximation as data did not reach a clear plateau. Group sizes were as follows: CTL-H01-H3 (0.1, 0.3, 1 and 3 mg/kg) (*n* = 10 per group, 1 animal excluded from 0.01, 0.3, and 1 mg/kg), CTL-H01-H2 (0.1, 0.3, 1, 3 and 6 mg/kg) (*n* = 6 per dose with 1 animal excluded from the 0.1 mg/kg group), CTL-P01-H1 (0.1, 0.3, 1, 3 and 6 mg/kg) (n = 6 per group, 2 animals and 1 animal excluded from the 1 mg/kg and 6 mg/kg groups, respectively), CTL-G01-H8 (0.03, 0.1, 0.3, 1 and 3 mg/kg) (n = 6 per group with 1 animal excluded from 0.03, 0.1, 0.3, and 1 mg/kg), CTL-G03-H2 (0.03, 0.1, 0.3, 1 and 3 mg/kg) (n = 6 per group with 1 animal excluded from 1 mg/kg), and CTL-X02-H1 (0.1, 0.3, 1, and 2 mg/kg) (n = 10 per group, 1 animal excluded from 0.01 and 2 mg/kg). Although CTL-P01.H1 appeared to produce a dose dependent increase in immobility time, this effect did not appear to reach a plateau and consequently, the data were not fit. LogED50 values determined for pure Δ^9^-THC and cannabis chemovar extracts are summarized in Table 3. One-way ANOVA followed by Dunnett’s multiple comparisons test revealed no significant differences between the logED50 for pure Δ^9^-THC and the logED50s for cannabis chemovar extracts (*P* = 0.90, F(5, 214) = 0.33).

One-way ANOVA followed by Tukey’s multiple comparisons test of the different groups of vehicle treated animals revealed no differences (*P* = 0.66, F(5, 37) = 0.66). Thus, relative maximum effects of cannabis chemovars were approximated by normalizing the effects at 3 mg/kg (with the exception for CTL-X02.H1 which was compared at the highest dose of 2 mg/kg). Using a one-way ANOVA followed by Dunnett’s multiple comparisons test, CTL-H01.H3 and CTL-X02.H1 revealed a significant difference in maximum effect. Other cannabis chemovar extracts did not produce any significant effects (Table 3). None of the chemovar extracts appeared to produce marked sedation. In general, those chemovars with higher levels of CBD relative to Δ^9^-THC did not markedly affect logED_50_ values determined in this in vivo assay. However, in the presence of elevated concentrations of the other non-Δ^9^-THC cannabinoid constituents (Table 2) in the chemovar, there may be added pharmacological activity that requires further study.

#### Effects of cannabis chemovar extracts on the standard hot plate assay

**Fig. 4:**
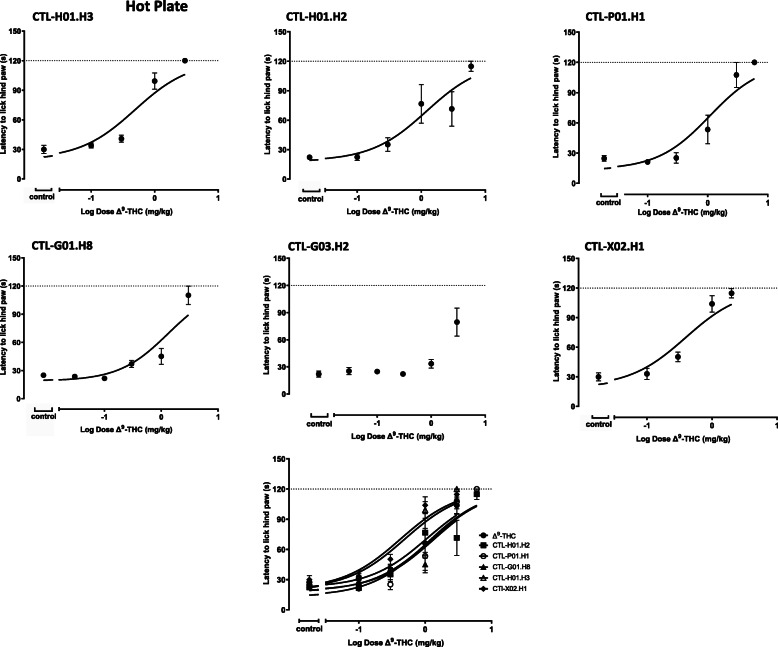
The effects of cannabis cultivar extracts were examined in the standard hot plate assay 5–10 min post-dose. Mice were intravenously dosed with control (vehicle) or CTL-H01-H3 (0.1, 0.3, 1 and 3 mg/kg) (n = 10 per group), CTL-H01-H2 (0.1, 0.3, 1, 3 and 6 mg/kg) (n = 6 per group), CTL-P01-H1 (0.1, 0.3, 1, 3 and 6 mg/kg) (n = 6 per group with 1 animal excluded from 6 mg/kg), CTL-G01-H8 (0.03, 0.1, 0.3, 1 and 3 mg/kg) (n = 6 per group), CTL-G03-H2 (0.03, 0.1, 0.3, 1 and 3 mg/kg) (n = 6 per group) or CTL-X02-H1 (0.1, 0.3, 1, and 2 mg/kg) (n = 10 per group) cannabis cultivar extract. The curves have been summarized on the same set of axes to facilitate visual comparisons. Data are presented as the mean ± SEM. Data were fit using a 3 parameter log (agonist) vs. response non-linear regression model. Dosing was based on the Δ^9^-THC content of the cannabis cultivar extract, as analyzed by HPLC

All cannabis chemovar extracts evaluated in the hot plate assay produced dose dependent analgesia (Fig. 4). Dose-response curves for chemovars may only be an approximation as data did not reach a clear plateau. Group sizes were as follows: CTL-H01-H3 (0.1, 0.3, 1 and 3 mg/kg) (*n* = 10 per group), CTL-H01-H2 (0.1, 0.3, 1, 3 and 6 mg/kg) (*n* = 6 per group), CTL-P01-H1 (0.1, 0.3, 1, 3 and 6 mg/kg) (n = 6 per group with 1 animal excluded from 6 mg/kg), CTL-G01-H8 (0.03, 0.1, 0.3, 1 and 3 mg/kg) (n = 6 per group), CTL-G03-H2 (0.03, 0.1, 0.3, 1 and 3 mg/kg) (n = 6 per group), CTL-X02-H1 (0.1, 0.3, 1, and 2 mg/kg) (n = 10 per group). Although CTL-G03.H2 appeared to produce dose dependent analgesia, the effect did not reach a plateau and consequently, the data were not fit to a dose response model. LogED50 values were calculated as an “ethical ED50” (eED50) because a true maximum could not be reached due to an ethical cut-off that was imposed to prevent injury to the animals (Table 3). One-way ANOVA followed by Dunnett’s multiple comparisons test for the logeED50 values indicated no significant differences between pure Δ^9^-THC and cannabis chemovar extracts (*P* = 0.004, F(5, 230) = 3.629).

One-way ANOVA followed by Tukey’s post hoc test indicated no differences between any of the vehicle groups (*P* = 0.59, F(5, 39) = 0.75). Thus, relative maximum effects of cannabis chemovars were approximated by normalizing the effects at 3 mg/kg (with the exception for CTL-X02.H1 which was compared at the highest dose of 2 mg/kg). One-way ANOVA followed by Dunnett’s multiple comparisons test revealed no differences between the maximum effect of Δ^9^-THC and any of the chemovars. However, because a cut-off threshold was used, pure Δ^9^-THC and all cannabis chemovar extracts tested were constrained to a maximum of 120 s. As a result, potential differences between chemovars and pure Δ^9^-THC may have been obscured by the threshold (Table 3).

## Discussion

This study is an attempt to develop a standardized process, using in vivo pharmacological methods, to assay chemovar extracts containing different concentrations of cannabinoids and their impact on potential therapeutic utility. These studies were conducted to define the pharmacological profile of a series of commercially available cannabis chemovar extracts after acute intravenous administration to mice using two validated assays which define components of the standard cannabinoid tetrad of studies used to characterize behavioral aspects of cannabinoid CB_1_ receptor activation (Fride et al. 2006). The concept of the conduct of appropriate pharmacological studies with quantifiable evaluation of cannabinoid content using well validated non-clinical models should be considered by the Cannabis industry. Data from such studies would better substantiate appropriate therapeutic use of the specific type of chemovar.

Prior to use in these studies, each cannabis chemovar extract was chemically characterized to profile major cannabinoid constituents. Providing an understanding of the pharmacological activity of cannabis chemovar extracts subsequently provides information on pharmacodynamic relationships since numerous phytocannabinoids are known to be present in the cannabis chemovar extract (Morales et al. 2017; El-Alfy et al. 2010; Haney et al. 2005). However, unlike Δ^9^-THC and CBD, little is known about the pharmacological activity of these cannabinoid constituents (Vann et al. 2008) since the primary focus has been to evaluate the profiles of Δ^9^-THC and CBD. Recently, Health Canada implemented the Cannabis Act to allow patients reasonable access to cannabis for medical purposes offering the potential benefit of administering cannabinoids as a group rather than individually. However, the safety pharmacology and toxicology assessments required for cannabis or cannabis extracts remain undefined.

While over 120 cannabinoids have been identified in cannabis, Δ^9^-THC and CBD are the major phytocannabinoids. Δ^9^-THC is the main component primarily responsible for the changes in behavior, cognition and perception associated with consumption. It is the substance responsible for altering consciousness, producing euphoria and relaxation but chronic use causes changes in memory, cognitive deficiencies, psychosis and dependence (Hood, 2018; Rice and Cameron 2018). Unlike Δ^9^-THC, CBD lacks the associated intoxicating effects when administered (Borgelt et al. 2013). GW Pharmaceuticals developed Epidiolex, a liquid formulation of pure plant-derived CBD, for Dravet’s syndrome, a severe form of childhood epilepsy (Corroon and Kight 2018). Thus, rather than use pure components of cannabis we have selected to characterize the natural cannabis chemovar extract and evaluate its efficacy profile in vivo.

The tail suspension test is a simple, objective method developed to produce ‘behavioral-desperation’ in animals which is manifest as periods of agitation (intense activity) and waiting behavior (immobility) (Steru et al. 1985; Porsolt et al. 1978). The assay is validated by an assessment of a diverse range of drugs with distinct pharmacological profiles and the findings are reproducible (Cryan et al. 2005). Two chemovars, CTL-H01.H3 and CTL-X02.H1, significantly increased the immobility time in the tail suspension assay. This is an intriguing finding as the chemovars exhibit vastly different cannabinoid profiles. At this time, it is unclear what accounts for the greater effects in the tail suspension assay compared to pure Δ^9^-THC, but according to Table 2, it is possible that THCV and THCA contributed to this effect, as both compounds were present in CTL-H01.H3 and CTL-X02.H1 chemovars. These results further support that THCV and THCA have effects on the CNS. Our results suggest that it will be important to report, in addition to the level of ∆^9^-THC, the level of THCV and THCA in cannabis extracts. This may allow the medical practitioners to better adjust the treatment, to increase the potential antidepressant effect while considering the potential psychoactive effects of a cannabis extract on patients. CBC (> 40 mg/kg, i.p.) has been shown to have some dose-dependent reduction in immobility in this model when administered as an individual cannabinoid component (El-Alfy et al. 2010). Although qualitatively, an increase in duration of immobility was observed with other chemovars, no significant differences were detected relative to pure Δ^9^-THC. However, we cannot rule out the possibility of a type II error and studies with larger group sizes should be undertaken to further explore the effect of cannabis chemovars and pure Δ^9^-THC in the tail suspension assay.

In this study, no cannabis chemovar extracts, regardless of cannabinoid profile, exhibited different logED50 or maximum values from pure Δ^9^-THC in the hot plate assay. Cannabis chemovar extracts were given equal dose ranges of Δ^9^-THC to examine the influence of the other phytocannabinoids present in the mixture. However, non-Δ^9^-THC cannabinoids did not appear to affect the potency of Δ^9^-THC in the assays tested in this study. It has been reported previously in the literature that CBD potentiates the analgesic effects of Δ^9^-THC, but that did not seem to be the case in this study (Varvel et al. 2006; Borgen et al. 1973). Although high levels of minor cannabinoids such as CBG, CBDV, and CBN relative to Δ^9^-THC to were present in some chemovars, we were unable to demonstrate that they enhanced analgesic potency. Unless the minor cannabinoids were equipotent with Δ^9^-THC, their pharmacological activity is likely obscured by the higher concentrations of Δ^9^-THC present in the selected cannabis chemovars. Δ^9^-THC has been characterized in this assay and produces dose-dependent antinociception (an increase in reaction time). Both pure Δ^9^-THC and the evaluated cannabis chemovar extracts produced analgesic responses with generally comparable ED_50_ values regardless of the Δ^9^-THC, CBD and cannabinoid constituent concentrations. Interestingly, CBD has been shown to allosterically modulate the CB_1_ receptor and limit the response of Δ^9^-THC (Borgen et al. 1973), but CBD did not show any detectable pharmacological activity for the study variables examined in these studies.

It is important to note the logED50 values may not be accurate as the data did not reach a clear and unequivocal plateau when fit to a dose response curve. Additionally, a cut-off threshold being imposed in the hot plate assay truncates the true efficacy of the tested material. The inability to obtain a true maximum would also alter the logED50 values in the hot plate assay. It would be prudent to test the cannabis chemovar extracts in other pain assays without an imposed cut-off threshold to obtain a true maximum value.

In summary, these studies show that when the acute intravenous effects of each cannabis chemovar were compared using two established assays, i.e., the tail suspension and hot plate, the majority of chemovars produced a similar pharmacological profile. However, two chemovars with starkly different cannabinoid profiles increased the maximum effect in the tail suspension assay compared to pure Δ^9^-THC. This raises the possibility that cannabinoids co-administered with Δ^9^-THC may modulate its effects. It is not clear at this time which components in the chemovars account for the different effects, but raises the possibility that different chemovars may produce different effects. Further studies are required to better understand the contribution of different cannabinoids in modulating the effects of Δ^9^-THC. It is important to note that these comparisons are preliminary and we urge future studies to investigate these possible differences in greater detail. Although humans are most likely to smoke cannabis chemovar material, these intravenous studies show that the extracts, even at high doses, can be used with a greater assurance of safety versus tolerability and safety issues associated with current analgesics, particularly opioids. However, further comprehensive testing is needed to characterize additional chemovars and the individual cannabinoid content in those chemovars in order to establish a safety database. Furthermore, data from the totality of such studies would assist in the optimization of the balance of Δ^9^-THC content to that of CBD and related cannabinoids to provide pain relief that may be individually tailored to a particular acute or chronic disease condition or specific to a patient requiring personalized pain control.

## Conclusion

Two of the tested chemovars produced pharmacological effects in the tail suspension assay that differed from Δ^9^-THC and the other cannabis chemovars. This finding suggests that the effects of cannabis chemovars may be influenced by their cannabinoid profile and provides evidence to support the entourage hypothesis. Currently, it is unclear which individual cannabinoids are responsible for the effect and may be a result of the combination of cannabinoids. Further studies are required to elucidate the cannabinoids or cannabinoid combinations responsible for enhancing the tail suspension effect. It is crucial to obtain cannabis chemovars which are low in Δ^9^-THC and CBD while containing high concentrations of the minor cannabinoids to understand the role of the minor cannabinoids in cannabis.

## Data Availability

The datasets used and/or analyzed during the current study are available from the corresponding author on reasonable request.

## Abbreviations

CBC: Cannabichromene
CBD: Cannabidiol
CBG: Cannabigerol
CBDV: Cannabidivarin
Δ^9^-THC: Δ9-tetrahydrocannabinol
